# Commentary: Primary Transcripts of microRNAs Encode Regulatory Peptides

**DOI:** 10.3389/fpls.2016.01436

**Published:** 2016-09-22

**Authors:** Shuo Lv, Lixia Pan, Guodong Wang

**Affiliations:** Key Laboratory of Ministry of Education for Medicinal Plant Resource and Natural Pharmaceutical Chemistry, National Engineering Laboratory for Resource Developing of Endangered Chinese Crude Drugs in Northwest of China, College of Life Sciences, Shaanxi Normal UniversityXi'an, China

**Keywords:** miRNA-encoded peptide (miPEP), pri-miRNA, long noncoding RNA (lncRNA), Arabidopsis, gene regulation

Noncoding RNAs (ncRNAs), including microRNAs (miRNAs) and long noncoding RNAs (lncRNAs), were assumed to be incapable of encoding proteins (Mercer et al., 2009; Rogers and Chen, 2013; Patil et al., 2014). miRNAs that derived from the primary miRNAs (pri-miRNAs) play crucial roles in post-transcriptional gene regulation by either repressing translation or guiding the degradation of complementary mRNA targets (Rogers and Chen, 2013). Transcriptome and high-throughput sequencing analyses have revealed a large number of ncRNAs in various organisms (Kapusta and Feschotte, 2014). It was found that ncRNAs were implicated in a variety of biological processes, including plant growth and development, and responses to environmental stresses (Mercer et al., 2009; Zhang et al., 2013; Kapusta and Feschotte, 2014).

Increasing evidence from both plants and animals has revealed that previously annotated lncRNAs have the capacity to encode small peptides (Ruiz-Orera et al., 2014; Lauressergues et al., 2015). In mammals, myoregulin (MLN), a lncRNA-encoded micropeptide, was reported to function in controlling muscle performance (Anderson et al., 2015). More specifically, MLN finely regulated calcium (Ca^2+^) uptake through physical interaction with sarcoplasmic reticulum Ca^2+^ ATPase (SERCA). The removal of MLN in mice resulted in enhanced Ca^2+^ handling and improved exercise performance (Anderson et al., 2015). A second lncRNA-encoded peptide, termed dwarf open reading frame (DWORF), has been shown to enhance SERCA activity and Ca^2+^ load by displacing the SERCA inhibitors and mitigating their inhibitory activity (Nelson et al., 2016). Meanwhile, in plants ~30,000 ncRNAs have been identified with over 1700 transcripts designated as ncRNAs in Arabidopsis alone (Liu et al., 2015). The functionally characterized plant lncRNA-encoded peptides comprise *ENOD40* (*Early nodulin 40*) that is required for plant-bacteria symbiotic interaction, *IPS1* (*Induced by phosphate starvation1*) that is implicated in phosphate uptake, *LDMAR* (*Long-day-specific male-fertility-associated RNA*) that controls photoperiod-sensitive male sterility, and *COOLAIR* and *COLDAIR* that influence Arabidopsis flowering time by affecting *FLC* transcription (Zhang et al., 2013).

Pri-miRNAs have been recently reported to harbor short open reading frames (ORFs) that encode regulatory peptides, termed miRNA-encoded peptides (miPEPs), indicating that pri-miRNAs possess both protein-coding and non-coding roles (Lauressergues et al., 2015). The native expression of miPEPs could be detected using specific antibodies, and their expression patterns are the same as those of their corresponding miRNAs. Overexpression or exogenous application of two miPEPs, miPEP171b from *Medicago truncatula* and miPEP165a from Arabidopsis, enhanced the expression of their corresponding miRNAs, thereby potentiating the suppression of target genes involved in root development (Lauressergues et al., 2015). Collectively, this study revealed that miPEPs are functional peptides that could promote the accumulation of their associated pri-miRNAs and ultimately down-regulate target genes.

The identification of miPEPs is in line with increasing evidence that a large number of micropeptides were found to be encoded by previously unannotated short ORFs in lncRNAs (Ruiz-Orera et al., 2014; Lauressergues et al., 2015). An immediate question in future is to determine whether miPEPs exist in other organisms, and if so, how many of these miPEPs have a biological function? This further raises another question that with what approaches to detect and validate potential miPEPs. The existence of endogenous miPEPs have been experimentally demonstrated using immunoblot, GUS reporter analysis and overexpression studies for miPEP171b and miPEP165a (Lauressergues et al., 2015). The translation of pri-miR171b and pri-miR165a were also supported by ribosome profiling (Juntawong et al., 2014) although miPEP171b and miPEP165a have not been detected by mass spectrometry (Baerenfaller et al., 2008; Castellana et al., 2008). The identification of miPEPs by using computational prediction alone is challenging (Waterhouse and Hellens, 2015). As have been shown for the discovery of small ORFs (smORF)-encoded peptides (Saghatelian and Couso, 2015), a combination of approaches including high-throughput RNA sequencing (RNA-seq), ribosome profiling, proteomics and bioinformatic is also required for identification of putative miPEPs (Aspden et al., 2014; Juntawong et al., 2014; Prabakaran et al., 2014).

A survey of fifty Arabidopsis pri-miRNAs revealed the presence of at least one putative smORF encoding a peptide in each sequence (Lauressergues et al., 2015). Further investigation of these putative miPEPs revealed that they did not share a common signature, suggesting that the regulatory activity of each putative miPEP is likely specific for their associated miRNA as have been experimentally shown for several miPEPs including miPEP171b and miPEP165a (Lauressergues et al., 2015). A key unanswered question will be how these different miPEPs perform their biological function, and whether the activation of pri-miRNA transcription is a prevalent mechanism for all miPEPs. The lncRNA-encoded micropeptides exert either inhibitory or stimulatory effects on their target genes in mammals (Anderson et al., 2015; Nelson et al., 2016). However, it remains unexplored whether miPEPs exert a negative effect on the expression of their associated miRNAs. Furthermore, it is intriguing to study whether any undiscovered components are involved in miPEP-mediated expression regulation, and whether any unknown means, which in turn modulate the positive effect of miPEPs.

As another aspect, miPEP synthesis and miRNA maturation occur in two physically distinct domains of pri-miRNAs. However, it is unclear how pri-miRNAs simultaneously coordinate their coding and non-coding capacities on the fact that cytoplasmic translation of pri-miRNA and nuclear maturation of miRNAs concurred. Regarding the coding function of pri-miRNA, genome editing to obtain loss-of-function mutants, in addition to overexpression and exogenous application of synthetic peptides, is necessary to assess the function of miPEPs. It is known that many peptides, including CLE peptides, are subjected to post-translational modifications (Matsubayashi, 2011). However, whether miPEPs are post-translationally modified remains unclear. Additionally, considering their small size, it is of interest to investigate whether miPEPs are transported to mediate long distance signals similarly to that of those post-translationally modified peptides (Okamoto et al., 2013).

Because miPEPs specifically promote the transcription of their respective pri-miRNAs which result in down-regulation of target genes, they represent an efficient means for studying their corresponding miRNA families and improving yields in agronomical crops. Indeed, exogenous application of synthetic miPEP172c, which stimulates miR172c expression, eventually results in nodule formation in soybean (Couzigou et al., 2016). In this regard, miPEPs could be used as alternative tools to optimize agronomical traits of crops (Couzigou et al., 2015). However, one need aware that application of synthetic peptides in fields would be costly.

In conclusion, miPEPs identification highlights the dual function of pri-miRNAs which combine both protein-coding and non-coding capacities. Elucidating how miPEPs function will illuminate their important regulatory features and reveal an additional level of gene regulation.

## Author contributions

GW and SL conceived and wrote the manuscript; LP critically reviewed the manuscript.

### Conflict of interest statement

The authors declare that the research was conducted in the absence of any commercial or financial relationships that could be construed as a potential conflict of interest.
